# The Molecular Pathogenesis of Osteosarcoma: A Review

**DOI:** 10.1155/2011/959248

**Published:** 2011-04-13

**Authors:** Matthew L. Broadhead, Jonathan C. M. Clark, Damian E. Myers, Crispin R. Dass, Peter F. M. Choong

**Affiliations:** ^1^Department of Orthopaedics, Department of Surgery, University of Melbourne, St. Vincent's Hospital, SVHM, L3, Daly Wing, 35 Victoria Parade, Fitzroy VIC 3065, Australia; ^2^School of Biomedical and Health Sciences, Victoria University, St. Albans, VIC 3021, Australia; ^3^Sarcoma Service, Peter MacCallum Cancer Centre, East Melbourne, VIC 3002, Australia

## Abstract

Osteosarcoma is the most common primary malignancy of bone. It arises in bone during periods of rapid growth and primarily affects adolescents and young adults. The 5-year survival rate for osteosarcoma is 60%–70%, with no significant improvements in prognosis since the advent of multiagent chemotherapy. Diagnosis, staging, and surgical management of osteosarcoma remain focused on our anatomical understanding of the disease. As our knowledge of the molecular pathogenesis of osteosarcoma expands, potential therapeutic targets are being identified. A comprehensive understanding of these mechanisms is essential if we are to improve the prognosis of patients with osteosarcoma through tumour-targeted therapies. This paper will outline the pathogenic mechanisms of osteosarcoma oncogenesis and progression and will discuss some of the more frontline translational studies performed to date in search of novel, safer, and more targeted drugs for disease management.

## 1. Introduction

Osteosarcoma is a relatively uncommon cancer although it is the most common primary malignancy to arise from bone. While incidence is low, osteosarcoma predominately affects adolescents and young adults, and if untreated it is fatal. Despite modern treatment protocols that combine chemotherapy, surgery, and sometimes radiotherapy, the 5-year survival rate for patients diagnosed with osteosarcoma remains at 60%–70% [1]. Current treatments for osteosarcoma are associated with significant morbidity, and a period of rehabilitation may be required following surgery for osteosarcoma. Hence, there is a real need to optimise current treatment strategies and to develop novel approaches for treating osteosarcoma.

Traditionally, our understanding of osteosarcoma has been largely anatomical. Osteosarcoma arises most commonly in the metaphyseal region of long bones, within the medullary cavity, and penetrates the cortex of the bone to involve the surrounding soft tissues. A pseudocapsule forms around the penetrating tumour [2]. Histologically, osteosarcoma is characterised as a highly cellular tumour composed of pleomorphic spindle-shaped cells capable of producing an osteoid matrix. Current standards for staging and surgical resection rely on this anatomical knowledge [3]. However, recent developments in molecular biology have provided insight into the molecular pathogenesis of osteosarcoma. Through the identification of tumour pathways and specific mediators of osteosarcoma progression, novel approaches for targeting osteosarcoma are being developed. This paper will review our current understanding of the molecular pathogenesis of osteosarcoma.

## 2. Pathogenesis

### 2.1. Bone Growth and Tumorigenesis

Osteosarcoma has a predilection for developing in rapidly growing bone. A number of studies have established a correlation between the rapid bone growth experienced during puberty and osteosarcoma development [4, 5]. Fifty-six percent of all osteosarcomas present around the knee [2]. The epiphyseal growth plates of the distal femur and proximal tibia are responsible for a great deal of the increase in height that occurs during puberty. Additionally, the peak age of osteosarcoma development is slightly earlier for females, an observation that may be explained by the relatively earlier growth spurt experienced by girls [6]. There is a male:female ratio of 1.5 : 1 for osteosarcoma, and patients affected by the disease are taller compared to the normal population of the same age group [7]. Patients affected by Paget's disease, a disorder characterised by both excessive bone formation and breakdown, also have a higher incidence of osteosarcoma [2].

### 2.2. Environmental Factors

Physical, chemical, and biological agents have been suggested as carcinogens for osteosarcoma. Among these, the role of ultraviolet and ionising radiation is the best established. The initial pathogenic link between radiation exposure and osteosarcoma was noted in female radium dial workers who applied radium to watch faces to make them luminescent [8]. However, radiation exposure is implicated in only 2% of cases of osteosarcoma [9] and is not thought to play a major role in paediatric disease. An interval of 10–20 years between exposure and osteosarcoma formation has been observed [10]. When radiotherapy is used in children as a treatment agent for a solid tumour, 5.4% develop a secondary neoplasm, and 25% of these are sarcomas [11].

The chemical agents linked to osteosarcoma formation include methylcholanthrene and chromium salts [12], beryllium oxide [13], zinc beryllium silicate [14], asbestos, and aniline dyes [15]. Previously, a viral origin had been suggested for osteosarcoma. This stemmed from the detection of simian virus 40 (SV40) in osteosarcoma cells. However, the presence of SV40 in these cells was later concluded to be the result of presence of SV40 viral units as contamination in the polio-virus vaccine that these patients had received [16, 17]. Studies evaluating the role of SV40 in the pathogenesis of mesothelioma have suggested that detection of SV40 in human cancers may in fact be due to laboratory contamination by plasmids containing SV40 sequences [18, 19].

### 2.3. Chromosomal Abnormalities

A number of chromosomal and genetic syndromes have been linked to osteosarcoma. Osteosarcoma has been reported in patients with Bloom syndrome, Rothmund-Thompson syndrome, Werner syndrome, Li-Fraumeni syndrome, and hereditary retinoblastoma [15]. Bloom, Rothmund-Thompson, and Werner [20] syndromes are characterised by genetic defects in the RecQ helicase family. DNA-helicases are responsible for separation of double-stranded DNA prior to replication [21, 22]. Mutations in these genes confer a higher risk of multiple malignancies.

A recent study of pretherapeutic biopsy specimens has identified amplifications of chromosomes 6p21, 8q24, and 12q14, as well as loss of heterozygosity of 10q21.1, as being among the most common genomic alterations in osteosarcoma. Furthermore, it was concluded that patients carrying these alleles had a poorer prognosis [23]. Numerical chromosomal abnormalities associated with osteosarcoma include loss of chromosomes 9, 10, 13, and 17 as well as gain of chromosome 1 [24].

### 2.4. Tumour Suppressor Gene Dysfunction

When human cells are exposed to environmental insults, such as those discussed above, somatic DNA may be damaged. Such DNA damage may not necessarily give rise to a malignant cell line, as there are a number of tumour-suppressor mechanisms in place. These mechanisms may either repair the DNA damage or induce apoptosis of these cells. The p53 and retinoblastoma (Rb) genes are well-known tumour-suppressor genes. However, tumour suppressor genes may themselves become mutated, resulting in the loss of their protective function. As a result, additional somatic mutations may accumulate, giving rise to a cell line that replicates without restraint. Mutations in both the p53 and Rb genes have been proven to be involved in osteosarcoma pathogenesis [6].

The p53 gene is mutated in 50% of all cancers and 22% of osteosarcomas [24]. DNA damage results in phosphorylation of p53, which is constitutively inhibited by Mdm2. Phosphorylation allows p53 dissociation from Mdm2. p53 exerts its tumour-suppressor effects via the activation of proapoptotic Bax and p21. The latter binds and inactivates G_1_/S-Cdk and S-Cdk complexes, causing arrest of the cell cycle in G_1_ [25]. 

Recently, p53 mutations have been shown to result in impaired DNA repair mechanisms and disrupted antiangiogenesis activity [26]. For osteosarcoma, the prototypical condition of p53 mutation is Li-Fraumeni syndrome. This syndrome is characterised by an autosomal dominant mutation of p53 leading to the development of multiple cancers including osteosarcoma [27]. Li-Fraumeni syndrome and germ-line mutations of p53 in osteosarcomas are rare, however [28], and in many osteosarcoma cell lines, a mutation in the first intron of the p53 gene occurs [29] though other point mutations have also been reported [30].

While p53 has been implicated in the oncogenesis of osteosarcoma, it is unclear whether p53 mutation or loss may affect tumour behaviour. Using the p53-null SaOS-2 osteosarcoma cell line, Ganjavi et al. [31] showed that adenoviral-mediated gene transfer of wild-type p53 resulted in reduced cell viability and increased sensitivity to chemotherapeutic agents. A recent study published by Hu et al. [32] showed that p53 expression was higher in low Rosen grade osteosarcomas (Rosen grade 1: <50% necrosis; grade 2: 50%–90% necrosis; grade 3: >90% necrosis; grade 4: 100% necrosis; grade 1 + 2 = low-grade; grade 3 + 4 = high grade). p53 expression correlated with reduced metastatic disease and improved survival for these patients. p53 mutation has also been shown to be more common in high-grade conventional osteosarcomas versus low grade central osteosarcomas [33]. However, other studies differ such as that of Lonardo et al. [34], which found no relationship between p53 and histological grade. Univariate analysis performed by Park et al. [35] showed no correlation between survival and the p53 protein, while coexpression of p53 and P-glycoprotein was associated with a poorer prognosis. 

In addition to p53, the Rb tumour suppressor has also been implicated in the tumorigenesis of osteosarcoma. The Rb gene is critical to cell-cycle control, and inherited mutation of the Rb gene causes retinoblastoma syndrome, a condition that predisposes a patient to multiple malignancies including osteosarcoma. The Rb protein regulates the cell cycle by binding the transcription factor E2F. E2F is held inactive by Rb until the CDK4/cyclin D complex phosphorylates Rb. Mutations of Rb allow for the continuous cycling of cells [25]. 

Both germ-line and somatic mutations of Rb confer an increased risk of osteosarcoma. Loss of the Rb gene may even explain the familial risk of osteosarcoma [36]. However, it has yet to be determined whether Rb gene loss or suppression gives rise to more aggressive tumours with poorer prognosis. Loss of heterozygosity for Rb has been reported to confer both an improved and poorer prognosis for patients [37–40]. In terms of response to chemotherapeutic treatment, Iida et al. [41] showed that the SaOS-2 osteosarcoma cell line, lacking active Rb, was less sensitive to the growth-suppressing effect of methotrexate compared to cell lines with wild-type Rb gene. Further studies are warranted to investigate the role of Rb on chemosensitivity of osteosarcoma cells.

### 2.5. Transcription Factors

Transcription is the process of forming single-stranded messenger RNA (mRNA) sequences from double-stranded DNA. Transcription factors facilitate binding of promoter sequences for specific genes to initiate the process. While transcription is usually tightly regulated, deregulation may occur in osteosarcoma, as with other cancers. Excess production of transcription factors, or the production of a new overactive transcription factor, may result from gene rearrangement.

The activator protein 1 complex (AP-1) is a regulator of transcription that controls cell proliferation, differentiation, and bone metabolism. AP-1 is comprised of Fos and Jun proteins, products of the c-fos and c-jun proto-oncogenes, respectively. Fos and Jun are found to be significantly upregulated in high-grade osteosarcomas compared with benign osteoblastic lesions and low-grade osteosarcomas [42, 43] and are associated with the propensity to develop metastases [44]. Fos and Jun double-transgenic mice are found to develop osteosarcomas with a higher frequency than c-Fos only transgenic mice [45]. Most recently, Leaner et al. [46] showed that inhibition of AP-1-mediated transcription caused reduced migration, invasion, and metastasis in a murine model of osteosarcoma. Another approach has been to target the Jun component of AP-1. The DNA enzyme Dz13 cleaves human c-Jun mRNA and is capable of inhibiting osteosarcoma growth and progression in a clinically relevant murine model when delivered by nanoparticle vector [47].

Myc is a transcription factor that acts in the nucleus to stimulate cell growth and division. Myc amplification has been implicated in osteosarcoma pathogenesis and resistance to chemotherapeutics. Overexpression of Myc in bone marrow stromal cells leads to osteosarcoma development and loss of adipogenesis [48]. Myc is amplified in U2OS osteosarcoma cell-line variants with the highest resistance to doxorubicin, and gain of Myc was found in SaOS-2 methotrexate-resistant variants [49]. Additionally, Myc has been examined as a therapeutic target for osteosarcoma. Downregulation of Myc enhanced the therapeutic activity of methotrexate against osteosarcoma cells [50]. Adenovirus-mediated transfection with the antisense Myc fragment led to cell-cycle arrest and enhanced apoptosis in the MG-63 osteosarcoma cell line [51]. Using a conditional transgenic mouse model, Arvanitis et al. [52] showed that Myc inactivation caused proliferative arrest and promoted differentiation in osteosarcoma. Additionally, using positron emission tomography (PET), these tumours exhibited reduced metabolic activity as demonstrated by reduced uptake of [^18^F]-fluorodeoxyglucose ([^18^F]-FDG).

### 2.6. Growth Factors

Osteosarcoma cells produce a range of growth factors that exert autocrine and paracrine effects. Dysregulated expression of growth factors such as transforming growth factor (TGF), insulin-like growth factor (IGF), and connective tissue growth factor (CTGF) leads to the accelerated proliferation of cells. Growth factor receptors may be overexpressed and constitutively activated. Signal transduction associated with these receptors may also be overactivated.

Transforming growth factor beta (TGF-*β*) proteins are a large family of dimeric proteins secreted by cells. Like many other growth factors, they influence a wide variety of cell process such as differentiation, proliferation, apoptosis, and matrix production. Bone morphogenic proteins (BMPs) make up a large component of the TGF-*β* family. High-grade osteosarcomas are found to express TGF-*β*1 in significantly higher amounts than low-grade osteosarcomas [53]. Navid et al. [54] examined the autocrine role of TGF-*β* on two osteosarcoma cell lines, demonstrating a 30%–50% reduction in growth when osteosarcoma cells were cultured in the presence of TGF-*β*-blocking antibody. Smad activation was implicated downstream of TGF-*β* with an inability to phosphorylate the Rb protein. Most recently, Hu et al. [55, 56] have shown an association between increased susceptibility and metastasis of osteosarcoma with TGFR1 variants, TGFBR1*6A, and Int7G24A. 

IGF (insulin-like growth factor)-I and IGF-II are growth factors that are often overexpressed by osteosarcomas. These ligands bind corresponding receptors such as IGF-1R, leading to activation of the PI3K and MAPK transduction pathways. This, then, supports cell proliferation and inhibition of apoptosis [57]. The growth-stimulating effect of IGF has been targeted for osteosarcoma. Lentivirus-mediated shRNA targeting IGF-R1 enhanced the chemosensitivity of osteosarcoma cells to docetaxel and cisplatin [58]. The use of monoclonal antibodies targeting IGF-R1 was also effective in enhancing antitumour response [59, 60]. 

Connective tissue growth factor (CTGF) is related to a number of proteins in the CCN family (CTGF/Cyr61/Cef10/NOVH). This protein family appears to act via integrin signalling pathways [61] and, like TGF-*β*, has a diverse range of functions including adhesion, migration, proliferation, survival, angiogenesis, and differentiation. Nishida et al. [62] showed that CTGF is a potent stimulator for the proliferation of SaOS-2 cells, leading to increased expression of type I collagen, alkaline phosphatase, osteopontin, and osteocalcin, markers for bone cell differentiation and maturation. A related protein, CCN3, was found to be overexpressed in osteosarcoma and associated with a worse prognosis [63].

Parathyroid hormone (PTH), parathyroid hormone-related peptide (PTHrP), and the receptor (PTHR1) have been implicated in the progression and metastasis of osteosarcoma. PTHrP was discovered as the humoral factor associated with tumour metastasis and hypercalcaemia [64]. The role of PTHrP and PTHR1 in osteoclast signalling will be discussed later. In terms of direct effects on osteosarcoma cells, when HOS osteosarcoma cells were overexpressed with PTHR1, increased proliferation, motility, and invasion through Matrigel were observed [65]. Gagiannis et al. [66] recently showed that PTHrP confers chemoresistance in osteosarcoma by blocking signalling via p53, death-receptor and mitochondrial pathways of apoptosis. PTHrP downregulated expression of proapoptotic Bax and PUMA and upregulated antiapoptotic Bcl-2 and Bcl-xl. Berdiaki et al. [67], using MG-63 and SaOS-2 osteosarcoma cell lines, showed that PTH peptides enhanced osteosarcoma cell migration through the regulation of hyaluron metabolism. However, a previous study showed that overexpression of PTHrP in a murine osteoblastic osteosarcoma cell line reduced cell proliferation by 80% [68]. Further studies are required to determine the prognostic significance of PTH/PTHrP/PTHR1 signalling in osteosarcoma.

### 2.7. Osteosarcoma Cell Proliferation, Apoptosis, and Anchorage-Independent Growth

Cancer cells are relatively resistant to apoptosis, and this ability to avoid elimination contributes to the ability of osteosarcoma cells to proliferate without restriction. Apoptosis consists of initiation and execution phases. During initiation, enzymes responsible for the cleavage of vital cellular proteins, known as caspases, are activated. Execution refers to the actual process of hydrolysis performed by activated caspases. Both extrinsic and intrinsic pathways regulate the initiation phase. The extrinsic pathway is a death receptor-initiated pathway, while the intrinsic pathway relies on increased mitochondrial permeability. Both proapoptotic and antiapoptotic factors interact with these pathways, and these have been discussed in a previous review [69].

Anoikis is a form of apoptosis that is induced when cells are no longer attached to a basement membrane or matrix. This is of particular interest in osteosarcoma given the propensity of osteosarcoma cells to detach from matrix components and to metastasise. Osteosarcoma cells are resistant to anoikis and proliferate despite deranged cell-cell and cell-matrix attachments. This resistance to anoikis is termed anchorage-independent growth (AIG).

**Figure 1:**
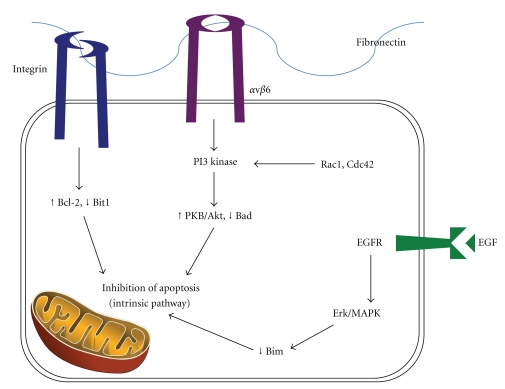
Pathways disrupting anoikis.

The pathways causing anoikis disruption and leading to anchorage-independent growth are complex. They involve interactions between integrin signalling, Rho GTPases, PI3 kinase, and PKB/Akt activation, along with many key components of the intrinsic and extrinsic apoptosis pathways (Figure 1). For example, when normal cells adhere to surrounding matrix via integrin-fibronectin binding, the Bcl-2 inhibitor Bit1 is suppressed allowing Bcl-2 to prevent apoptosis via the intrinsic pathway [70]. Another pathway involves the exchange of integrin subunits resulting in the production of abnormal integrins, such as *α*v*β*6, which can upregulate PI3 kinase function [71]. PI3 kinase can then activate PKB/Akt which inhibits the proapoptotic factor Bad, leading to cancer cell survival [72]. Rho GTPases such as Rac1 and Cdc42 can also upregulate PI3 kinase with similar consequences [73]. Increased epidermal growth factor-receptor (EGF/EGFR) binding with subsequent extracellular signal-regulated kinase (Erk)/microtubule-associated protein kinase (MAPK) signalling leading to inhibition of Bim has also been described [74]. This suppresses cell death, as Bim would normally act to increase mitochondrial outer membrane permeability allowing release of cytochrome *c* and then the activation of executioner caspases.

### 2.8. Tumour Angiogenesis

Tumour angiogenesis is essential for sustained osteosarcoma growth and metastasis. Without a supporting vasculature, osteosarcoma cells would be unable to obtain the nutrients and oxygen necessary for proliferation. Metastasis to the lungs and bone, the most common sites for osteosarcoma spread, also relies on the formation and maintenance of blood vessels. Radiation therapies, while compromising tumour cells, also destroy the vascular component of tumours and block the supply of nutrients. So, radio- and chemotherapies act by these dual actions. This aspect is discussed below.

A balance between pro-angiogenic and antiangiogenic factors regulates angiogenesis, and this balance is tipped towards the favour of neovascularisation by tissue hypoxia, acidosis, oncogene activation, and loss of tumour suppressor gene function. A hypoxic and acidotic microenvironment exists around proliferating osteosarcoma cells, and these conditions stimulate deubiquitination of von Hippel Lindau protein. Von Hippel Lindau protein releases hypoxia-inducible factor-1*α* (HIF-1*α*), allows HIF-1*α* to bind to the promoter region of the vascular endothelial growth factor (VEGF) gene [75], and upregulats it. TGF-*α*, and fibroblast growth factor (FGF) may also upregulate VEGF [76].

VEGF is the best-characterised pro-angiogenic factor, and it stimulates the processes of endothelial cell proliferation, migration, and blood vessel maturation. A number of different VEGF molecules exist (VEGF-A through to VEGF-E), and these proteins bind to VEGF receptors (VEGFR1-3) [77]. VEGF-A has the broadest angiogenic effect. Upon VEGF-A binding to VEGFR2, a number of divergent signalling pathways are initiated [77]. Nitric oxide (NO) is released by endothelial cells, leading to vasodilation and increased vascular permeability [78]. Endothelial cell proliferation and cycling are stimulated via phospholipase C*γ* (PLC*γ*), protein kinase C (PKC), and the c-Raf-MEK-MAPK cascades [77]. Rearrangement of the actin cytoskeleton, necessary for endothelial cell migration occurs via phosphorylation of T cell-specific adapter (TSAd) and interaction with Src, another protein kinase [79]. The net result of all these changes is the formation of an immature, irregular, and leaky vascular network.

The immature and inefficient nature of the vessels so produced facilitates feedback loops for further vessel formation. Upregulation of HIF-1*α* and VEGF [80] again occurs as the leaky vasculature is unable to meet the metabolic demands of the proliferating osteosarcoma cells. Additionally, VEGF upregulates matrix metalloproteinase (MMP) and plasmin activity [81]. These proteases break down extracellular matrix, which releases any VEGF combined with heparin proteoglycan in the matrix. VEGF also induces antiapoptotic factors Bcl-2, and survivin, ensuring ongoing endothelial proliferation [82]. In addition to VEGF, the proliferating tumour cells release a number of other pro-angiogenic factors. These include FGF, platelet-derived growth factor (PDGF), angiopoietin1 (Ang1), and ephrin-B2 [83, 84].

While it is known that osteosarcoma is a relatively vascular tumour, the prognostic significance of this is yet to be determined. There have been studies suggesting both a correlation [85, 86] and lack of association [87] between VEGF expression and osteosarcoma microvascular density and metastases at diagnosis. This may relate to a greater tumour dependence on functionally mature vessels. One study that demonstrated a survival advantage associated with increased osteosarcoma microvascular density [88] attributed this advantage to improved tissue penetration by chemotherapeutic agents.

As previously mentioned, angiogenesis is regulated by the balance between pro-angiogenic and antiangiogenic factors. Antiangiogenic proteins such as thrombospondin 1, TGF-*β* [89], troponin I, pigment epithelial-derived factor (PEDF) [90], and reversion-inducing cysteine rich protein with Kazal motifs (RECK) [91] are downregulated in osteosarcoma. These antiangiogenic molecules are particularly important for embryogenesis and physiological processes such as wound healing and menstruation; however, they also play a protective mechanism against osteosarcoma progression. For example, troponin I and PEDF are expressed predominately within the avascular zones of the cartilaginous growth plate [92, 93] and are likely to contribute to growth plate resistance to osteosarcoma invasion from a typical metaphyseal location. In addition to inhibiting angiogenesis, PEDF exerts direct effects on osteosarcoma cells. Ek et al. [94, 95] have demonstrated apoptosis induction in osteosarcoma cell lines treated with PEDF. Also, in a murine model of orthotopic osteosarcoma, tumour volume was reduced by PEDF, which was associated with reduced microvascular density. There was decreased tumour metastases and reduced size of metastatic tumours in lung.

### 2.9. Cell Adhesion and Migration

Osteosarcoma is a highly metastatic tumour, and pulmonary metatases are the most common cause of death. The metastatic sequence involves the detachment of osteosarcoma cells from the primary tumour, adhesion to the extracellular matrix, local migration and invasion through stromal tissue, intravasation, and extravasation. The ability of osteosarcoma cells to metastasise by such a pathway relies on complex cell-cell and cell-matrix interactions.

The extracellular matrix is composed of various protein fibrils and growth factors. The proteins include fibronectin, collagens, proteoglycans, and laminins. Osteosarcoma cells may also produce matrix proteins. The extracellular matrix provides a developing tumour with a supporting scaffold and facilitates blood vessel formation. Osteosarcoma cells adhere to matrix components via cell-surface receptors. These receptors are more than just a physical point of attachment; they also provide a link between matrix proteins and the cytoskeleton. The principle receptor proteins are the integrins, which bind to the matrix protein fibronectin. There are 24 different integrin heterodimer molecules consisting of different *α* and *β* subunits [96]. 

The integrins also play a role in cell signaling, particularly in pathways critical to cell migration. Integrin-binding proteins such as talin become associated with the cytoplasmic domain and act, via adaptor proteins such as vinculin, paxillin, and *α*-actin, for the upregulation of protein kinases [97]. The key enzymes involved here are focal adhesion kinase (FAK), protein kinase C (PKC), PI3 kinase, Src, and the RhoA GTPases. 

The relative activities of these enzymes underlie conformational changes in cell architecture. For example, there is a shifting balance between two of the RhoA GTPases: Rac1 and RhoA. High Rac1 expression suppresses RhoA and induces the formation of membrane ruffles. These membrane changes facilitate cell spreading and migration [98]. Conversely, high RhoA with low Rac1 leads to membrane retraction. These two processes are coordinated such that in cell migration, the leading edge of the cell is demonstrating actin polymerisation and lamellipoedia, while the trailing edge is undergoing actin disassembly. Inhibition of RhoA pathways has been shown to reduce osteosarcoma cell migration and invasion [99]. 

In general, cells migrate towards ligand-dense matrix and towards more rigid matrix [100], indicating a constant intracellular response to extracellular adhesion and tension. Tumour stroma is more rigid than normal connective tissue matrix, and this generates integrin clustering, activation of intracellular signalling pathways, decreases cell-to-cell contacts, and stimulates tumour growth [101]. 

The ezrin protein also has a role in cell-cell interactions, signal transduction, linkage between actin filaments, and cell membrane receptors such as CD44, which binds hyaluronan in the extracellular matrix. When ezrin is overexpressed, it is associated with an increase in metastasis [102]. Increased ezrin expression in paediatric osteosarcoma patients is associated with reduced disease-free intervals, and downregulation of ezrin expression in a mouse model of human osteosarcoma has been shown to reduce pulmonary metastasis [103].

### 2.10. Tumor Invasion

Invasion of the surrounding tissues by osteosarcoma also involves degradation of the extracellular matrix. Matrix metalloproteinases (MMPs) are principally involved in the breakdown of the extracellular matrix, although roles in tumour angiogenesis have also been established.

MMPs are a family of zinc-dependent endopeptidases that are involved in a range of physiological processes including inflammation, wound healing, embryogenesis, and fracture healing. In normal tissues, MMPs are regulated by natural inhibitors such as tissue inhibitors of MMPs (TIMPs), RECK, and *α*2 macroglobulin [104]. In the setting of osteosarcoma, MMPs break down extracellular collagens, facilitating both tumour and endothelial cell invasion. MMPs may be designated as gelatinases, collagenasesm, or stromeolysins. Gelatinases break down denatured collagens and type IV collagen. Collagenases break down type I, type II, and type III collagen, and stromeolysins break down proteoglycan (found in articular cartilage), type III, type IV (in basement membranes), and type V collagen, as well as casein and fibronectin [105]. 

In addition to clearing a pathway for invading osteosarcoma cells, the role of MMPs in angiogenesis has already been mentioned. Remodelling of vessel walls by MMPs gives rise to a thin and leaky vascular network that allows passage of tumour cells into the bloodstream [106]. Furthermore, MMP-9 releases VEGF stored within the extracellular matrix [107], and VEGF is able to upregulate MMP-2 [108]. The specific importance of the gelatinases MMP-2 and MMP-9 to tumour progression has been delineated in an *in vivo* study, where combined MMP-2/MMP-9 deficiency in mice significantly impaired tumour angiogenesis and invasion [109].

The urokinase plasminogen activator (uPA) system is the other key regulator of osteosarcoma invasion, which interacts with MMPs. The ligand uPA binds to its receptor uPAR to become active. Once activated, uPA cleaves plasminogen to plasmin. Plasmin breaks down the extracellular matrix but also activates pro-MMPs. A cascade of activation is hence established [110, 111]. The role of the uPA-uPAR system is well established in osteosarcoma pathogenesis. An inverse relationship between uPA levels and survival time has been demonstrated [112]. Downregulation of uPAR in an *in vivo* osteosarcoma model resulted in reduced primary tumour growth and fewer metastases [113].

### 2.11. Osteoclast Function

**Figure 2:**
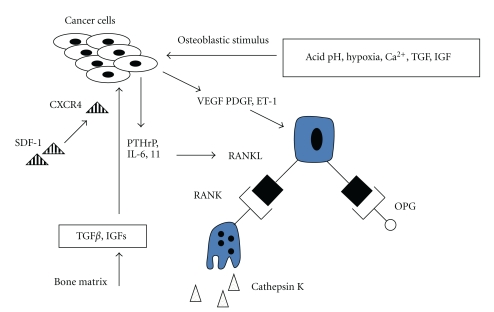
Vicious cycle of osteolysis by osteoclasts.

Osteosarcoma invasion of bone relies on interactions between the bone matrix, osteosarcoma cells, osteoblasts, and osteoclasts (Figure 2). Osteoclasts are the principle bone-resorbing cells, and the substantial osteolysis exhibited by some osteosarcomas is the direct result of increased osteoclastic activity. During the initial stages of osteosarcoma invasion, growth factors such as TGF-*β* are released from the degraded bone matrix and act on osteosarcoma cells, stimulating the release of PTHrP, interleukin-6 (IL-6) and interleukin-11 (IL-11) [114, 115]. These cytokines then stimulate osteoclasts, facilitating further invasion and release of proresorptive cytokines.

Osteoblasts are, in fact, mediators in this process of bone resorption. Osteosarcoma cells release endothelin-1 (ET-1), VEGF, and PDGF in response to the hypoxic and acidotic conditions. These factors have predominantly osteoblast-stimulatory functions [116, 117]. PTHrP and IL-11 also act on osteoblasts, stimulating increased expression of receptor activator of nuclear factor *κ*B ligand (RANKL). RANKL is a key mediator of osteoclast differentiation and activity, and osteosarcoma cells have been noted to produce RANKL independently [118]. 

RANKL activates osteoclasts through binding to RANK on the osteoclast surface. RANK expression is under control of cytokines IL-1, IL-6, IL-8, tumour necrosis factor-*α* (TNF-*α*), PTHrP, and TGF-*α* [119]. Receptor-ligand binding initiates a cascade of events through binding of TRAF-6, leading to activation of both NF*κ*B and MAPK pathways, with a resulting increase in nuclear factor of activated T-cells (NFATc1) activity. RANK/RANKL also activates the c-Fos component of AP-1, resulting in additional NFATc1 upregulation. NFATc1 is thus a common end-point for effecting transcription of genes involved in osteoclast activity and maturation [120]. 

Activated osteoclasts release proteases to resorb the nonmineralised components of bone. Cathepsin K (Cat K) is a cysteine protease selectively produced by osteoclasts for breakdown of collagen I, osteopontin, and osteonectin [121]. Cat K is also produced by some cancer cells to aid invasion [122]. This protease is essential for osteoclast function in normal bone remodelling and also in pathological states of osteolysis. For patients with high-grade metastatic osteosarcoma, low Cat K levels at the time of diagnosis confers a better prognosis [123].

c-Src is a nonreceptor tyrosine kinase present within osteoclasts [124] and is involved in pathways regulating cell growth, survival, and migration [125]. Osteoclast survival occurs through c-Src mediating phosphatidylinositol 3-kinase and TRAF-6 interaction, with resulting Akt/mTOR (mammalian target of rapamycin) pathway activation and then inhibition of caspase-3 [126]. Podosome assembly, vesicle transport, secretion of proteases, and organisation of microtubules are all regulated by c-Src pathway activity [127]. For osteosarcoma, inhibition of c-Src induces apoptosis and inhibits invasion *in vitro*. Primary tumour volume in a murine model of osteosarcoma was also reduced by c-Src inhibition [128].

Osteoclast pathways of differentiation, maturation, and activation have potential as therapeutic targets. Inhibition of bone resorption at the tumour-bone interface may lead to reduced local invasion by osteosarcoma. The central role that RANKL plays in osteoclast function makes it a particularly attractive target. Osteoprotegerin (OPG) is a soluble decoy receptor for RANKL and strongly suppresses osteoclast differentiation both *in vitro* and *in vivo* [129]. OPG gene therapy has been applied to a murine model of osteosarcoma and successfully suppressed osteolytic activity. There were a reduced number of osteoclasts associated with tumours, leading to reduced local osteosarcoma progression and improved survival [130].

## 3. Summary and Future Directions

Osteosarcoma is a relatively uncommon malignancy, with an overall incidence of 5 cases per million persons per year. However, among childhood malignancies, osteosarcoma is the eighth most common. Only leukaemias, lymphomas, and neurological malignancies are more common. Osteosarcoma accounts for 8.9% of cancer-related deaths in children and carries an overall 5-year survival rate of 60%–70% [131]. However, being a disease that affects patients in the prime of their lives, incidence and survival rates do not accurately reflect the true burden of this disease. The burden to patients and the community is particularly high as our current treatments combine chemotherapy, often disabling surgery, and prolonged periods of rehabilitation. The disability-adjusted life year (DALY) was put forward by the World Health Organisation (WHO) as a measure of overall burden of disease. It is the number of years lost due to disability, poor health, or premature death. For sarcomas, an average of 17 life years per patient is lost, compared to 6.5 for bowel, lung, and breast cancers. For this reason, the treatment of osteosarcoma is a major public health issue.

Ottaviani and Jaffe [131] recently published a review of the epidemiology of osteosarcoma. Death rates for osteosarcoma are declining by a small 1.3% per year. Indeed, there has been no significant improvement in prognosis for patients with osteosarcoma since the advent of multiagent chemotherapy. Prior to chemotherapy, the overall survival rate for osteosarcoma was a dismal 20% [132]. However, the challenges we now face are paradoxically the result of our application of modern chemotherapeutics. Resistance to chemotherapy and the recurrence of disease, commonly in the form of pulmonary metastases despite successful surgical resection, are the two greatest challenges we face in regards to the development of therapies for osteosarcoma.

Our understanding of the molecular basis of osteosarcoma has advanced considerably over recent decades. The processes involved in osteosarcoma oncogenesis have been outlined above, and it is our hope that a molecular understanding of the disease will lead to targeted treatment of osteosarcoma. As is evident from the discussion above, there are potentially multiple targets, and we must identify and develop those with the most promise. Therapeutic approaches may not target osteosarcoma cells themselves but may seek to intervene in the complex biology between osteosarcomas cells, osteocytes, osteoblasts, osteoclasts, and even endothelial cells. Indeed, some of the more promising therapeutic agents developed exploit multiple tumorigenic pathways. For example, the potent antiangiogenic pigment epithelium derived factor (PEDF) inhibits the supporting vasculature of the developing tumour whilst also inhibiting proliferation, invasion, and metastasis of osteosarcoma cells [94, 133–135]. Similarly, reversion-inducing cysteine rich protein with Kazal motifs (RECK) has been shown to reduce microvascular density, tumour invasion, and metastasis independently [136].

In this paper we have sought to outline the molecular pathogenesis of osteosarcoma with some reference to potential therapeutic targets currently under investigation. The genetic basis of osteosarcoma has been presented and discussed, along with the role of key transcription factors and growth factors. The processes of osteosarcoma cell proliferation, apoptosis, adhesion, invasion, and metastasis represent potential biological targets for treating osteosarcoma. Osteoclast and endothelial cells may also be targeted. However, the study of pathogenic mechanisms is in itself not enough. Translational studies are critical if an effective treatment for osteosarcoma is to arise from this understanding of osteosarcoma biology. The past decade has revealed a great deal about osteosarcoma pathogenesis, and only with further translational studies will, we see which of the many potential targets and combination of therapies prove to be the most effective in treatment of this debilitating tumour.
